# Supplementary material for: Regional and demographic variations of Carotid artery Intima and Media Thickness (CIMT): A Systematic review and meta-analysis
Source: PLoS One. 2022 Jul 12;17(7):e0268716. doi: 10.1371/journal.pone.0268716 (PMC9275715; doi:10.1371/journal.pone.0268716)
Supplement: S1 File — (DOCX) [file pone.0268716.s001.docx]

**S 1 File: Search strategy for PubMed**

1. "carotid intima media thickness"[Title/Abstract] OR "CIMT"[Title/Abstract] OR "intima media thickness"[Title/Abstract] OR "intima media thickening"[Title/Abstract] OR "intimal media thickness"[Title/Abstract] OR "intima media complex"[Title/Abstract] OR "intimal medial complex"[Title/Abstract] OR "wall thickening"[Title/Abstract] OR "artery wall thickness"[Title/Abstract] OR (("tunica media"[MeSH Terms] OR ("tunica"[All Fields] AND "media"[All Fields]) OR "tunica media"[All Fields]) AND "diagnostic imaging"[Title/Abstract]) OR (("tunica intima"[MeSH Terms] OR ("tunica"[All Fields] AND "intima"[All Fields]) OR "tunica intima"[All Fields]) AND "diagnostic imaging"[Title/Abstract])

2. "Carotid"[Title/Abstract] OR "carotid arteries"[Title/Abstract] OR "carotid artery"[Title/Abstract]

3. "Cardiovascular diseases"[Title/Abstract] OR "coronary artery disease"[Title/Abstract] OR "CAD"[Title/Abstract] OR "myocardial infarction"[Title/Abstract] OR "ischaemic heart disease"[Title/Abstract]

4. "atherosclero*"[Title/Abstract] OR "atherosclerosis"[Title/Abstract]

5. "ultrasound"[Title/Abstract] OR "ultrasonograph"[Title/Abstract] OR "sonograph*"[Title/Abstract] OR "ultrasonography"[Title/Abstract]

6. "Adults"[Title/Abstract] OR "age"[All Fields]) AND "over 40 years"[Title/Abstract]) OR "older"[Title/Abstract] OR "Healthy"[Title/Abstract] OR "CHD"[Title/Abstract]

7. (("carotid arteries"[MeSH Terms] OR ("carotid"[All Fields] AND "arteries"[All Fields]) OR "carotid arteries"[All Fields] OR "carotid"[All Fields] OR "carotids"[All Fields] OR "carotidal"[All Fields] OR "carotide"[All Fields] OR "carotideal"[All Fields]) AND ("intimae"[All Fields] OR "tunica intima"[MeSH Terms] OR ("tunica"[All Fields] AND "intima"[All Fields]) OR "tunica intima"[All Fields] OR "intima"[All Fields] OR "intimas"[All Fields]) AND ("culture media"[Pharmacological Action] OR "culture media"[MeSH Terms] OR ("culture"[All Fields] AND "media"[All Fields]) OR "culture media"[All Fields] OR "media"[All Fields] OR "media s"[All Fields] OR "medias"[All Fields]) AND ("diabete"[All Fields] OR "diabetes mellitus"[MeSH Terms] OR ("diabetes"[All Fields] AND "mellitus"[All Fields]) OR "diabetes mellitus"[All Fields] OR "diabetes"[All Fields] OR "diabetes insipidus"[MeSH Terms] OR ("diabetes"[All Fields] AND "insipidus"[All Fields]) OR "diabetes insipidus"[All Fields] OR "diabetic"[All Fields] OR "diabetics"[All Fields] OR "diabets"[All Fields])) OR "Hypertention"[All Fields] OR ("dyslipidaemias"[All Fields] OR "dyslipidemias"[MeSH Terms] OR "dyslipidemias"[All Fields] OR "dyslipidaemia"[All Fields] OR "dyslipidemia"[All Fields])

8. ("carotid arteries"[MeSH Terms] OR ("carotid"[All Fields] AND "arteries"[All Fields]) OR "carotid arteries"[All Fields] OR "carotid"[All Fields] OR "carotids"[All Fields] OR "carotidal"[All Fields] OR "carotide"[All Fields] OR "carotideal"[All Fields]) AND ("intimae"[All Fields] OR "tunica intima"[MeSH Terms] OR ("tunica"[All Fields] AND "intima"[All Fields]) OR "tunica intima"[All Fields] OR "intima"[All Fields] OR "intimas"[All Fields]) AND ("culture media"[Pharmacological Action] OR "culture media"[MeSH Terms] OR ("culture"[All Fields] AND "media"[All Fields]) OR "culture media"[All Fields] OR "media"[All Fields] OR "media s"[All Fields] OR "medias"[All Fields]) AND "BMI"[All Fields]

9. ("carotid intima media thickness"[MeSH Terms] OR ("carotid"[All Fields] AND "intima media"[All Fields] AND "thickness"[All Fields]) OR "carotid intima media thickness"[All Fields] OR ("carotid"[All Fields] AND "intima"[All Fields] AND "media"[All Fields] AND "thickness"[All Fields]) OR "carotid intima media thickness"[All Fields]) AND ("alcohol s"[All Fields] OR "alcoholate"[All Fields] OR "alcoholates"[All Fields] OR "alcoholic s"[All Fields] OR "alcoholics"[MeSH Terms] OR "alcoholics"[All Fields] OR "alcoholic"[All Fields] OR "alcoholism"[MeSH Terms] OR "alcoholism"[All Fields] OR "alcoholisms"[All Fields] OR "alcoholism s"[All Fields] OR "alcoholization"[All Fields] OR "alcohols"[MeSH Terms] OR "alcohols"[All Fields] OR "ethanol"[MeSH Terms] OR "ethanol"[All Fields] OR "alcohol"[All Fields])

10. ("carotid intima media thickness"[MeSH Terms] OR ("carotid"[All Fields] AND "intima media"[All Fields] AND "thickness"[All Fields]) OR "carotid intima media thickness"[All Fields] OR ("carotid"[All Fields] AND "intima"[All Fields] AND "media"[All Fields] AND "thickness"[All Fields]) OR "carotid intima media thickness"[All Fields]) AND ("smoke"[MeSH Terms] OR "smoke"[All Fields] OR "smoke s"[All Fields] OR "smoked"[All Fields] OR "smokes"[All Fields] OR "smoking"[MeSH Terms] OR "smoking"[All Fields] OR "smokings"[All Fields] OR "smoking s"[All Fields])

11. ("carotid intima media thickness"[MeSH Terms] OR ("carotid"[All Fields] AND "intima media"[All Fields] AND "thickness"[All Fields]) OR "carotid intima media thickness"[All Fields] OR ("carotid"[All Fields] AND "intima"[All Fields] AND "media"[All Fields] AND "thickness"[All Fields]) OR "carotid intima media thickness"[All Fields]) AND ("exercise"[MeSH Terms] OR "exercise"[All Fields] OR "exercises"[All Fields] OR "exercise therapy"[MeSH Terms] OR ("exercise"[All Fields] AND "therapy"[All Fields]) OR "exercise therapy"[All Fields] OR "exercise s"[All Fields] OR "exercised"[All Fields] OR "exerciser"[All Fields] OR "exercisers"[All Fields] OR "exercising"[All Fields])

12. ("carotid intima media thickness"[MeSH Terms] OR ("carotid"[All Fields] AND "intima media"[All Fields] AND "thickness"[All Fields]) OR "carotid intima media thickness"[All Fields] OR ("carotid"[All Fields] AND "intima"[All Fields] AND "media"[All Fields] AND "thickness"[All Fields]) OR "carotid intima media thickness"[All Fields]) AND (("medical history taking"[MeSH Terms] OR ("medical"[All Fields] AND "history"[All Fields] AND "taking"[All Fields]) OR "medical history taking"[All Fields] OR ("family"[All Fields] AND "history"[All Fields]) OR "family history of"[All Fields]) AND ("cancer health disparities"[Journal] OR "chd"[All Fields]))

13. ("carotid intima media thickness"[MeSH Terms] OR ("carotid"[All Fields] AND "intima media"[All Fields] AND "thickness"[All Fields]) OR "carotid intima media thickness"[All Fields] OR ("carotid"[All Fields] AND "intima"[All Fields] AND "media"[All Fields] AND "thickness"[All Fields]) OR "carotid intima media thickness"[All Fields]) AND ("air pollution"[MeSH Terms] OR ("air"[All Fields] AND "pollution"[All Fields]) OR "air pollution"[All Fields])

14. (("carotid intima media thickness"[MeSH Terms] OR ("carotid"[All Fields] AND "intima media"[All Fields] AND "thickness"[All Fields]) OR "carotid intima media thickness"[All Fields] OR ("carotid"[All Fields] AND "intima"[All Fields] AND "media"[All Fields] AND "thickness"[All Fields]) OR "carotid intima media thickness"[All Fields]) AND ("air pollution"[MeSH Terms] OR ("air"[All Fields] AND "pollution"[All Fields]) OR "air pollution"[All Fields])) OR (("carotid intima media thickness"[MeSH Terms] OR ("carotid"[All Fields] AND "intima media"[All Fields] AND "thickness"[All Fields]) OR "carotid intima media thickness"[All Fields] OR ("carotid"[All Fields] AND "intima"[All Fields] AND "media"[All Fields] AND "thickness"[All Fields]) OR "carotid intima media thickness"[All Fields]) AND ("air pollution"[MeSH Terms] OR ("air"[All Fields] AND "pollution"[All Fields]) OR "air pollution"[All Fields])) OR (("carotid intima media thickness"[MeSH Terms] OR ("carotid"[All Fields] AND "intima media"[All Fields] AND "thickness"[All Fields]) OR "carotid intima media thickness"[All Fields] OR ("carotid"[All Fields] AND "intima"[All Fields] AND "media"[All Fields] AND "thickness"[All Fields]) OR "carotid intima media thickness"[All Fields]) AND (("medical history taking"[MeSH Terms] OR ("medical"[All Fields] AND "history"[All Fields] AND "taking"[All Fields]) OR "medical history taking"[All Fields] OR ("family"[All Fields] AND "history"[All Fields]) OR "family history of"[All Fields]) AND ("cancer health disparities"[Journal] OR "chd"[All Fields])))

15. ("carotid intima media thickness"[MeSH Terms] OR ("carotid"[All Fields] AND "intima media"[All Fields] AND "thickness"[All Fields]) OR "carotid intima media thickness"[All Fields] OR ("carotid"[All Fields] AND "intima"[All Fields] AND "media"[All Fields] AND "thickness"[All Fields]) OR "carotid intima media thickness"[All Fields]) AND ("ethnical"[All Fields] OR "ethnically"[All Fields] OR "ethnicities"[All Fields] OR "ethnicity"[MeSH Terms] OR "ethnicity"[All Fields] OR "ethnic"[All Fields] OR "ethnics"[All Fields] OR "ethnology"[MeSH Subheading] OR "ethnology"[All Fields] OR "ethnology"[MeSH Terms])

16. ("carotid intima media thickness"[MeSH Terms] OR ("carotid"[All Fields] AND "intima media"[All Fields] AND "thickness"[All Fields]) OR "carotid intima media thickness"[All Fields] OR ("carotid"[All Fields] AND "intima"[All Fields] AND "media"[All Fields] AND "thickness"[All Fields]) OR "carotid intima media thickness"[All Fields]) AND ("educational status"[MeSH Terms] OR ("educational"[All Fields] AND "status"[All Fields]) OR "educational status"[All Fields] OR ("educational"[All Fields] AND "level"[All Fields]) OR "educational level"[All Fields])

17. ("carotid intima media thickness"[MeSH Terms] OR ("carotid"[All Fields] AND "intima media"[All Fields] AND "thickness"[All Fields]) OR "carotid intima media thickness"[All Fields] OR ("carotid"[All Fields] AND "intima"[All Fields] AND "media"[All Fields] AND "thickness"[All Fields]) OR "carotid intima media thickness"[All Fields]) AND ("occupant"[All Fields] OR "occupant s"[All Fields] OR "occupants"[All Fields] OR "occupational"[All Fields] OR "occupations"[MeSH Terms] OR "occupations"[All Fields] OR "occupation"[All Fields])

18. (("carotid intima media thickness"[MeSH Terms] OR ("carotid"[All Fields] AND "intima media"[All Fields] AND "thickness"[All Fields]) OR "carotid intima media thickness"[All Fields] OR ("carotid"[All Fields] AND "intima"[All Fields] AND "media"[All Fields] AND "thickness"[All Fields]) OR "carotid intima media thickness"[All Fields]) AND ("occupant"[All Fields] OR "occupant s"[All Fields] OR "occupants"[All Fields] OR "occupational"[All Fields] OR "occupations"[MeSH Terms] OR "occupations"[All Fields] OR "occupation"[All Fields])) OR (("carotid intima media thickness"[MeSH Terms] OR ("carotid"[All Fields] AND "intima media"[All Fields] AND "thickness"[All Fields]) OR "carotid intima media thickness"[All Fields] OR ("carotid"[All Fields] AND "intima"[All Fields] AND "media"[All Fields] AND "thickness"[All Fields]) OR "carotid intima media thickness"[All Fields]) AND ("educational status"[MeSH Terms] OR ("educational"[All Fields] AND "status"[All Fields]) OR "educational status"[All Fields] OR ("educational"[All Fields] AND "level"[All Fields]) OR "educational level"[All Fields])) OR (("carotid intima media thickness"[MeSH Terms] OR ("carotid"[All Fields] AND "intima media"[All Fields] AND "thickness"[All Fields]) OR "carotid intima media thickness"[All Fields] OR ("carotid"[All Fields] AND "intima"[All Fields] AND "media"[All Fields] AND "thickness"[All Fields]) OR "carotid intima media thickness"[All Fields]) AND ("ethnical"[All Fields] OR "ethnically"[All Fields] OR "ethnicities"[All Fields] OR "ethnicity"[MeSH Terms] OR "ethnicity"[All Fields] OR "ethnic"[All Fields] OR "ethnics"[All Fields] OR "ethnology"[MeSH Subheading] OR "ethnology"[All Fields] OR "ethnology"[MeSH Terms]))
