# Supplementary material for: Regional and demographic variations of Carotid artery Intima and Media Thickness (CIMT): A Systematic review and meta-analysis
Source: PLoS One. 2022 Jul 12;17(7):e0268716. doi: 10.1371/journal.pone.0268716 (PMC9275715; doi:10.1371/journal.pone.0268716)
Supplement: Attachment — Submitted filename: Responce to Reviewers.docx [file pone.0268716.s007.docx]

**Responses to the raised review points by the academic editor and reviewers**

**Academic editor:**

**Comment**

1. “Please ensure that your manuscript meets PLOS ONE's style requirements, including those for file naming.”

**Response**

Manuscript formatted according to PLOS ONE style requirements.

**Comment**

2. “We note that one or more of the authors is affiliated with the funding organization, indicating the funder may have had some role in the design, data collection, analysis or preparation of your manuscript for publication; in other words, the funder played an indirect role through the participation of the co-authors. If the funding organization did not play a role in the study design, data collection and analysis, decision to publish, or preparation of the manuscript and only provided financial support in the form of authors' salaries and/or research materials, please do the following:”

a. Review your statements relating to the author contributions, and ensure you have specifically and accurately indicated the role(s) that these authors had in your study. These amendments should be made in the online form.

b. Confirm in your cover letter that you agree with the following statement, and we will change the online submission form on your behalf: “The funder provided support in the form of salaries for authors [insert relevant initials], but did not have any additional role in the study design, data collection and analysis, decision to publish, or preparation of the manuscript. The specific roles of these authors are articulated in the ‘author contributions’ section.

**Response**

We selected option B. We include following statement in the cover letter. “The funder provided support in the form of salaries for author [VA], but did not have any additional role in the study design, data collection and analysis, decision to publish, or preparation of the manuscript. The specific roles of these authors are articulated in the ‘author contributions’ section.”

**Comment**

3. “In your Data Availability statement, you have not specified where the minimal data set underlying the results described in your manuscript can be found. PLOS defines a study's minimal data set as the underlying data used to reach the conclusions drawn in the manuscript and any additional data required to replicate the reported study findings in their entirety.”

**Response**

We included the link to accesses minimum data set in the manuscript. (Page 29)

**Comment**

4. “Please include captions for your Supporting Information files at the end of your manuscript, and update any in-text citations to match accordingly.”

**Response**

Captions for supporting information files are included at the end of manuscript. (Page 29)

**Reviewer 1:**

**Comment**

**Major comments**:

1. “Section “Method and analysis”, paragraph “Study selection“: According to this paragraph, titles and abstracts of the search results were screened against pre-specified criteria by two independent reviewers for study selection. Please add a description of the pre-specified criteria for study selection to the text.”

**Response**

Description of the pre-specified criteria for study selection was included in the method section. (Page 07)

**Comment**

2. “Section “Method and analysis”, paragraph “Study quality”: The quality of selected studies was assessed using the Quality Assessment of Diagnostic Accuracy Studies (QUADAS-2) criteria. It is important to assess the quality of reports in a consistent manner with other studies. The CONSORT statement and the STROBE statement represent 2 of the most widely accepted and used guidelines for accurate reporting and transparency. Therefore, it is recommended to additionally assess the quality of reports in reference to the CONSORT statement and the STROBE statement prior to including them in the analysis.”

**Response**

Summary of	STROBE statement is included as a supplementary table. (S2 Table. Summary of STROBE statement). [Description was added to page 9 to 12]. Our review had no randomized controlled trials. Therefore we did not assess our studies based on the CONSORT statement.

**Comment**

3. “Section “Method and analysis”, paragraph “Steps of Meta-analyses”: The meta-analyses were performed using random-effects models. Which model was used? Was the model based on inverse variance-weighted average method or weighted sum of z-scores?”

**Response**

We used the inverse variance-weighted average method. Correction has been done in the steps of meta-analysis section. (Page 09)

**Comment**

4. “Section “Method and analysis”, paragraph “Steps of Meta-analyses”: Due to the comparison of highly heterogeneous populations in different WHO regions with very divers individual risk profiles, there is a high chance of confounding. With regard to the extracted CIMT estimates, how did the model of the authors account for the risk of residual confounding? Were the estimates extracted from the primary studies confounder-adjusted or unadjusted data? The extraction process should be clarified. In addition, comparisons of adjusted and crude estimates allow insights into the importance of confounding. To reliably detect independent regional differences as well as potential influencing variables, the random-effects model should be adjusted for all known risk factors for cardiovascular disease, if possible from the data set.”

**Response**

Most of the primary studies reviewed had adjusted for selected confounders. We have included a separate column in table 1 to indicate the factors adjusted for in each study. As the estimates we used were already adjusted, we did not adjust for them again in the Meta analysis regression as we felt that it would be an over-adjustment. Also most studies had not adequately described their populations and it was not possible to adjust for them as the information that could be obtained gave country estimates which may not be reflective of populations in individual studies (eg: Caucasian populations etc.). However, we have presented crude and adjusted estimates for age, WHO regions, ultrasound methods and CHD group in table 3. (Page 23)

**Comment**

5. “Section “Method and analysis”, paragraph “Assessment of heterogeneity of studies”: In addition to the QUADAS-2 criteria, the Newcastle-Ottawa Scale should be applied to minimize risk of bias.”

**Response**

As suggested by the reviewers we have also used the Newcastle-Ottawa Scale for case control, cohort and cross sectional studies and the findings are included as supplementary files S3, S4 and S5, respectively. [Description was added to page 9 to 12]

**Comment**

6. “Section “Results”: What was the percentage of included subjects from grey literature and from published literature? What proportion of the total number of included patients is derived from each of the databases as a source? These numbers should be added to the results section as well as to the flow diagram in “Figure 1”.

**Response**

These have been included in the text as well as in Figure 1.

**Comment**

7. “Section “Discussion”: The discussion is mainly descriptive with an extended presentation of the results of the meta-analysis and its underlying primary studies. A reflection of possible underlying factors and differential population characteristics of the respective WHO regions would contribute to strengthen the discussion. Furthermore, general text flow and readability of the discussion should be significantly improved”.

**Response**

We have added an in-depth discussion on risk factors and different population characteristics to the discussion section. We have reorganised and re-written the discussion to improve the text flow and readability.

**Comments**

**Minor comments:**

1. “Section “Abstract”: The search strategy is not clearly described in the abstract. Therefore, adding a summarized description of the search strategy is recommended.”

**Response**

A summarized description of the search strategy has been included.

**Comment:**

2. “Section “Method and analysis”, paragraph “Search strategy”: The search strategy for PubMed should be specified in the text section of the search strategy paragraph. The separate box displaying the paragraph should be removed from the main manuscript.”

**Response**

The search strategy has been included as a supplementary file (S1 file).

**Comment**

3. “Section “Method and analysis”, paragraph “: Data extraction”: For articles based on the same population, the authors state that the ‘more comprehensive one’ was selected. Please add a precise description of the data extraction criteria.”

**Response**

A description was added to data extraction section. (Page 7 and 8)

**Comment**

4. “Section “Results”, “Table 3” and “Table 4”: “Table 3” and “Table 4” show mean CIMT values of different carotid segments by WHO region among patients with and without CHD. To provide a more focused illustration of the results, the content of “Table 3” and “Table 4” should be summarized in a single table.”

**Response:**

CIMT values of different carotid segments by WHO regions among patients with and without CHD were shown in table 2 and 3 which we have combined in to single table. (Table 2).

**Comment**

5. “Section “Results”: The text flow in the sections results is partially very tough. Therefore, it should be revised to ensure a more fluid presentation of the results.”

**Response:**

As suggested, we revised the text flow of the results section.

**Reviewer 2:**

**Major comments:**

**Comment**

1. “Regarding the conclusion, that there is a need to establish country specific CIMT cutoff values, I find it difficult to state this in light of the presented data. For my understanding in this study, differences between countries have not been investigated (since the results are based on WHO regions). As the authors also state in the discussion, there are many factors that affect CIMT, and there are known regional differences in the prevalence of those risk factors (e.g. BMI, hypertension, diabetes). Since reference values are based on studies on a healthy population, it would be interesting to look at geographical differences in comparing healthy cohorts. Is there a possibility to address this? Otherwise I would recommend to rewrite this part of the conclusion.”

**Response**

We agree with the reviewer regarding this comment. Unfortunately we are unable to do this at the moment as it is beyond the scope of this manuscript. We will attempt it later.

**Comment**

2. “Ethnical aspects: It is known that in regards to cardio- and cerebrovascular disease the ethnical background plays a detrimental role. The presented study seems to calculate the values of patients from countries, but irrespective of their ethnicity. Is there also a possibility to analyse the impact of the ethnical background?”

**Response**

Most studies included in this review had not documented the ethnic compositions of their samples. Although we attempted to match countries with published percentages of ethnicities, we did not pursue it further as we felt that there are major variations in ethnic compositions even within countries. We have discussed this.

**Comment**

3.“Geographical aspects: There is also a different burden of cardiovascular and cerebrovascular diseases within one WHO region, e.g. Northern Europe versus southern Europe. Some regions also seem to be underrepresented. Authors tried to apply statistical methods relativize this fact and they also comment this in the study limitations section, that WHO classification is also partly based on political aspects as well. Would it not be clinically more reasonable to compare trials of comparable quality using a classification that is only based on geographical aspects (even taking into account not to cover the whole globe)?”

**Response**

Yes, we agree with the reviewer. However, for reasons stated in the response for the earlier comment, we cannot do it. We will attempt it later.

**Comment**

4. “Time span: Authors have reviewed and compared data on CIMT in the time span from 1980 to 2020. Within 40 years, the spatial resolution of ultrasound systems has revolutionized and is still becoming more precise, and therefore the measurements of vessels and their segments are not entirely comparable. Furthermore, there are significant differences in measuring the CIMT manually and automatically. Further factors that have changed over the decades are methodical quality of trials, the quality of trial performance, and the presence of trial audits are not respected in the study and are very likely to influence the outcome. Would it be possible to analyze only studies with a similar technological standard? This would improve the value of this study, since it would minimize bias due to technical and methodical differences.”

**Response**

We have considered ultrasound systems in the meta-regression analysis as suggested by the reviewer. (Page 23)

**Comment**

5. “Search strategy seems to be representative according to selected keywords, but the heterogeneity of population and patients with co-morbidity is not reflected.”

**Response**

Main objective of our study was to evaluate the CIMT variation across WHO regions, age and sex. We did not assess the heterogeneity of population and patients with co-morbidity in this study. We are planning to conduct it in a future event.

**Minor Comments:**

**Comment**

6. “Line 59: word „diseases “is missing.”

**Response**

Added the word.

**Comment**

7. “Line 64: The sentence starting with “The current COVID-19 pandemic…” is not relevant to the presented review and I would suggest to delete it.”

**Response**

We deleted the sentence.
