# Supplementary material for: Regional and demographic variations of Carotid artery Intima and Media Thickness (CIMT): A Systematic review and meta-analysis
Source: PLoS One. 2022 Jul 12;17(7):e0268716. doi: 10.1371/journal.pone.0268716 (PMC9275715; doi:10.1371/journal.pone.0268716)
Supplement: Attachment — Submitted filename: Responce to Reviewers .docx [file pone.0268716.s008.docx]

**Responses to the raised review points by the reviewers**

**Reviewer 1:**

**Comment**

**Major comments**:

1. Section “Discussion”: How do the authors interpret the value of these findings? Because of the descriptive background of this analysis and the different adjustment for different risk factors regarding the included studies, it is indeed difficult to demonstrate an incremental value of the participant’s geographical region/country. Therefore, as already indicated by reviewer 2, the topic should be considered very cautiously in the discussion. Statements on causal relationships and incremental value of the variable region for risk prediction should therefore be avoided. Adapt more defensive wording regarding this relationship.

**Response**

We understand the concerns of the reviewer. We have made the following changes:

Abstract – we reworded and included the following sentences

Older persons and those having CHD group had significantly thicker CIMTs – results section (Line 54)

There is an association between CHD and CIMT values – conclusion (Line 57)

In the results section of the body of the manuscript the sentence was reworded as

“In the adjusted model, CHD group, WHO region and age were significantly associated with CIMT.” (Line 331)

In the discussion, the following sentence was added.

“Even though there is a clear association between CIMT and CHD its usability as a risk predictor for CHD needs to be further investigated. Approaches to prevention as well as screening of at-risk populations for CHD may need to consider regional variations of CIMT.” (Line 389 to 391)

The following is the conclusion of the manuscript

“CIMT among the non-CHD group varies between and within regions, and by age and sex. The mean CIMT values between non-CHD and CHD groups were significantly different within and between WHO regions possibly due to varying influences of modifiable and non-modifiable risk factors. CHD group had a significantly thicker mean CIMT after adjusting for age, WHO region and ultrasound machine used. Segment specific CIMT variations exist among regions.”(Line 455 to 459)

**Minor Comment**

1. Section “Results”, Table 1: “Summary of studies used in systematic review and meta-analysis, reporting demography: As requested, the authors added further information on the adjustment of the studies included in the analyses. However, the mention of "factors adjusted for" and "adjusted predictors of CIMT" seems repetitive. Therefore, the column "Adjusted predictors of CIMT" should be removed since all relevant information is already listed in the column "Factors adjusted for."

**Response**

We removed the column.

**Minor Comment**

2. Section “Method and analysis”: Grammar and spelling of the newly added text parts should be revised.

**Response**

Grammar and spelling has been revised.

**Minor Comment**

3. Section “Results”: Since the included studies and corresponding details are already listed in Table 1, there is no need to cite the respective studies again separately in the results section.

**Response**

As suggested, correction has been done in the result section by omitting the references.

**Reviewer 2:**

**Minor comments:**

1. The conclusion, that region or country specific CIMT values are important when developing risk assessment tools to screen at-risk population of CHD is not supported by the data presented, since in this study only differences in CIMT between WHO regions were examined and not their impact to a certain CV risk and significant confounding is possible. CIMT in fact may be important in the risk assessment, but the presented data do not fully validate the role of CIMT differences between WHO regions regarding CV risk. Furthermore, in my opinion the conclusion that CHD is a predictor for CIMT is clinically not sound. As for my understanding, the presented data show a clear association between CHD and CIMT and not necessarily that the presence of CHD predicts CIMT values.

Therefore, I would recommend to precise the conclusion according to the presented data which may help to improve the quality of the manuscript.

**Response**

As suggested by the reviewer, we have deleted the sentence that country-specific CIMT values are important for risk prediction of CHD.

We have reworded the relevant sentences that stated that CHD is a predictor of CIMT. Please see response to major comment by reviewer 1 above which gives all details of the corrections made.
